# Serum Total Bilirubin, not Cholelithiasis, is Influenced by *UGT1A1* Polymorphism, Alpha Thalassemia and β^s^ Haplotype: First Report on Comparison between Arab-Indian and African β^s^ Genes

**DOI:** 10.4084/MJHID.2015.060

**Published:** 2015-11-01

**Authors:** Said Y. Alkindi, Anil Pathare, Shoaib Al Zadjali, Vinodhkumar Panjwani, Fauzia Wasim, Hammad Khan, Pradeep Chopra, Rajagopal Krishnamoorthy, Salam Alkindi

**Affiliations:** 1 Ministry of Health, Muscat, Oman & McMaster University, Canada.; 2 Sultan Qaboos University Hospital, Muscat, OMAN.; 3 INSERM, U665, F-75015 Paris, France; Laboratoire d’Excellence GR-EX, Paris, France.; 4 Sultan Qaboos University, College of Medicine & Health Sciences, Muscat, OMAN.

## Abstract

**Background:**

We explored the potential relationship between steady state serum bilirubin levels and the incidence of cholelithiasis in the context of *UGT1A1* gene A(TA)nTAA promoter polymorphism in Omani sickle cell anemia (SCA) patients, homozygotes for African (Benin and Bantu) and Arab-Indian β^S^ haplotypes, but sharing the same microgeographical environment and comparable life style factors.

**Methods:**

136 SCA patients were retrospectively studied in whom imaging data including abdominal CT scan, MRI or Ultrasonography were routinely available. Available data on the mean steady state hematological/biochemical parameters (n=136), β^s^ haplotypes(n=136), α globin gene status (n=105) and *UGT1A1* genotypes (n=133) were reviewed from the respective medical records.

**Results:**

The mean serum total bilirubin level was significantly higher in the homozygous *UGT1A1*(AT)_7_ group as compared to *UGT1A1*(AT)_6_ group. Thus, not cholelithiasis but total serum bilirubin was influenced by UGT1A1 polymorphism in this SCA cohort.

**Conclusion:**

As observed in other population groups, the *UGT1A1* (AT)_7_ homozygosity was significantly associated with raised serum total bilirubin level, but the prevalence of gallstones in the Omani SCA patients was not associated with α thalassaemia, *UGT1A1* polymorphism, or β^s^ haplotypes.

## Introduction

Chronic hemolysis in sickle cell anemia (SCA), results in hyperbilirubinemia, as the water insoluble bilirubin needs to be enzymatically converted into water soluble bilirubin glucoronides, for its elimination through bile by uridine glucuronosyltransferase (UGT) enzyme.^1^,^2^ Chronic hyperbilirubinemia, thus over time, can lead to the formation of gall stones (cholelithiasis), a common complication in SCA.^1^–^3^ Its onset can be as early as 2 to 4 years, but the prevalence increases progressively with age.^4^,^5^

Inherited common sequence variations (polymorphisms) in the promoter region of the *UGT1A1* gene that encodes the UGT enzyme had originally been associated with Gilbert’s syndrome, a benign nonhemolytic hyperbilirubinemia in the absence of liver disease.^6^ The same variations had consistently been associated, in various population groups, with hyperbilirubinemia in several hemolysis-related clinical conditions *viz.* SCA, β thalassaemia and hereditary spherocytosis.^6^–^8^ These polymorphisms correspond to a simple sequence (TA) repeat number variation in the TATA promoter motif of *UGT1A1* gene and had been shown to affect its expression. The alleles differ in the number of repeats from 5 to 8 with (TA)^6^ allele being the common allele in Caucasians.^7^ There is an inverse correlation between the number of repeats and hepatic expression level of the *UGT1A1*gene on the one hand, and on the other, a direct correlation between the number of repeats and bilirubinemia.^9^,^10^ However, the relationship of *UGT1A1* polymorphism, both with hyperbilirubinemia and with the incidence of cholelithiasis is not that straightforward. Several inconsistencies raise the possibility that other factors (genetic, environmental or both) may modulate either the extent of hemolysis or the rate of gall stone formation or both^10^–^12^. Studies of factors that could affect the hemolysis in SCA such as α thalassaemia and HbF have also produced conflicting data.^10^–^12^ Other inconsistencies include the significantly lower prevalence of cholelithiasis in African SCA patients as compared to Jamaicans or African-Americans despite bearing similar African β^s^ haplotypes.^1^,^9^,^10^

In this regard, Haider et al., studying SCA patients from Kuwait, mostly bearing the homozygous Arab-Indian haplotypes and high frequency of alpha thalassaemia, report that the prevalence of gallstones was much higher than that reported for Nigerian children with African β^s^ haplotypes.^13^ This datum is intriguing given the known influence of alpha thalassemia in reducing hemolysis. Such population and geographical discrepancies in the incidence of cholelithiasis further highlight the possibility that differences in the environmental (dietary cholesterol/fibers, use of third generation cephalosporins), life style factors (fasting, smoking) and/or genetic factors other than *UGT1A1* may explain such inconsistencies. Omani SCA patients offer an exceptional opportunity to clarify some of the above mentioned issues, as both African and Arab-Indian β^s^ haplotypes in the homozygous state are found in significant numbers sharing similar life style and clinical interventional factors^14^. In this context, the present single center cross sectional study allows certain homogeneity in terms of clinical management/interventions.

This study investigates the influence of *UGT1A1* polymorphism, HbF level, β^s^ haplotypes and α thalassaemia on the steady state bilirubinemia and propensity to develop gall stones in Omani SCA patients.

## Patients and Methods

**Figure 1:**
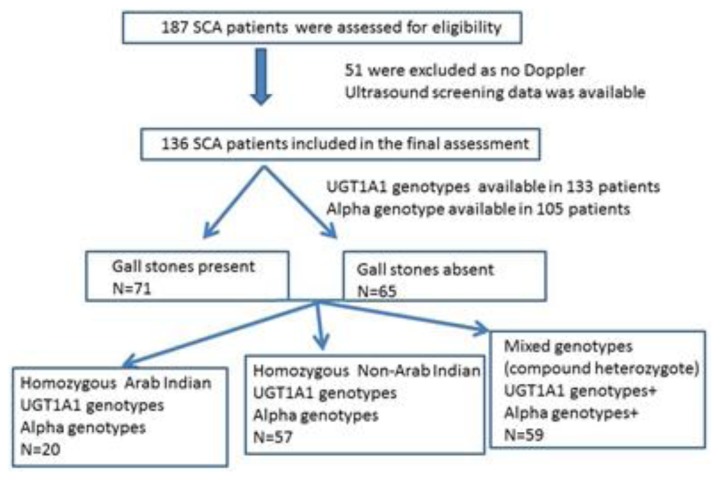
Enrollment of SCA patients and the evaluation categories.

The study patients were all from the hematology clinic at Sultan Qaboos University Hospital (SQUH). After getting approval from the hospital medical research and ethics committee and obtaining informed consent from patients or guardians, a total of 136 SCA patients were selected for whom imaging data (abdominal CT scan, MRI or Ultrasonography) performed as a routine study were available. (Figure 1). Information on patients who underwent cholecystectomy was also recorded. The presence of sickle cell mutation was also confirmed at the DNA level. The β^s^-globin gene cluster haplotype, α globin gene status, and *UGT1A1* polymorphism were determined as described earlier.^5^,^15^–^17^ DNA sequencing of the polymerase chain reaction (PCR)-amplified entire β-globin gene segment (including the promoter, all exons, and exon-intron junctions) was performed on an ABI PRISM 3100 Genetic Analyzer (Applied Biosystems, Foster City, CA, USA). Data on the mean steady state hematological/biochemical parameters (n=136), β^s^ haplotypes(n=136), α globin gene status (n=105) and *UGT1A1* genotypes(n=133) were reviewed retrospectively from the medical records and utilized for this analysis. 61(44.85%) of these were on stable hydroxyurea (HU) therapy.

### Statistical methods

Allele and genotype frequencies of the (TA)_n_ repeat were determined and tabulated. Deviations, if any, from the expected Hardy-Weinberg equilibrium, was calculated by the Chi square test. Differences in hematological data between different groups (*UGT1A1*, α thalassaemia and Sickle haplotype groups) of patients were assessed using Student’s t test and Chi square test. All the analysis was performed using STATA ver. 11.1 (StataCorp, College Station, TX, USA). A p value of <0.05 was considered as significant.

## Results

Tables 1a and 1b summarize the relevant demographic (sex, age), red blood cell (Hb, HbF, reticulocytes), current therapy with HU and biochemical (total serum bilirubin) parameters along with the prevalence of gall stone for the 136 study patients for whom the status of cholelithiasis was available. Non statistically significant differences were noted between males and females in any of these parameters; respectively, Hb 9.7±1.6 vs. 9.1±0.9g/dl, HbF 8.9±6.7 vs. 10.1±6.6% and total serum bilirubin 50.9±36.7 vs. 42.2±44 μmol/L. None below ten years had stones, but the cumulative percentage of stones peaked in the fourth decade to 70.5%. The mean total serum bilirubin level reached the maximum in the second decade while the incidence of gall stones in the fourth decade. Overall 77 patients were homozygotes for β^s^ haplotypes (20 - AI/AI & 57 - Ben/Ben or Ban/Ban) while all others were mixed haplotypes. In each decade age group, SCA patients with African haplotypes were more represented in percentage than those with the Arab-Indian haplotype.

In Table 2, the influence of the *UGT1A1* promoter polymorphism, stratified into three groups as described by Chaar V et al^9^ on the total serum bilirubin as well as on the prevalence of gall stones was examined along with the mean LDH, total Hb, HbF% and absolute reticulocyte count in 133 SCA patients. Two way comparisons show that the total serum bilirubin levels were significantly associated with *UGT1A1* polymorphism but not with the prevalence of gall stones. No statistically significant difference was noted among the *UGT1A1* genotype groups with respect to indicators of hemolysis (serum LDH, reticulocyte count, and Hb) and HbF level.

Since α thalassaemia can modulate the rate of hemolysis, we examined its influence on these parameters. We first analyzed if the α globin genotypes were comparable between the *UGT1A1* genotype groups. As expected and as shown in Table 3a, no difference was noted in the prevalence of α thalassaemia among the three *UGT1A1* groups (n=105). As shown in Table 3b the homozygous state for α thalassaemia (-α/-α genotype) was significantly associated with all parameters examined (except the rate of gall stones) as compared to individuals with four alpha globin genes. Such difference persisted for total serum bilirubin and LDH in a two way comparison between subjects with –α/-α genotype and –α/αα genotype. The differences were restricted to total Hb and absolute reticulocyte count in the comparison between -α/αα and αα/αα genotypes.

When a similar analysis was carried out for all these parameters based on homozygous β^s^ genotypes (Arab-Indian, Benin, and Bantu), we did not observe any difference between the Benin and Bantu homozygotes (data not shown) but both together were significantly different from the homozygotes for Arab-Indian haplotype. This allowed us to combine both the Benin and Bantu homozygotes into a single “African β^s^ genotype” group for statistical comparisons against the Arab-Indian β^s^ genotype.

First we checked for the alpha thalassemia status between the Arab-Indian and African β^s^ genotype groups, and as shown in Table 4a, although overall prevalence of alpha thalassemic genotype was higher among African β^s^ genotype group, the difference failed to reach statistical significance (p>0.05, chi square test). Data in Table 4b show that the subjects homozygous for the Arab-Indian β^s^ genotype are distinct from those having the African β^s^ genotypes in terms of bilirubin and hemolysis-related factors (lower total serum bilirubin, serum LDH and absolute reticulocyte count but a higher Hb and HbF%).

## Discussion

Geographical differences in the prevalence of cholelithiasis are now well recognized, but the factors influencing the rate of hemolysis, heme catabolism, and hepatic transport system seem to be multiple. So, the interplay between genetic and environmental (dietary and other xenobiotics including therapeutic drugs) factors seem to be complex and generate inconsistencies in various reports. We reasoned that if such analysis is performed in an SCA patient population group harboring both Arab-Indian [AI] and African β^s^ haplotypes but sharing comparably similar socio-cultural and life style factors, the influence of β^s^ genotype on cholelithogenesis may become more evident.

### Gender effect

Adekile et al. reported that the female gender influenced the serum bilirubin level in SCA patients from North America.^18^ We did not find such effect either on mean serum bilirubin level or on the incidence of gallstone. This discrepancy with Adekile study may be related to the higher HbF level observed in the female patients as compared to males, but in our study, although the HbF levels in females were relatively higher than the males, they were not significantly high enough to reach a statistical significance of p<0.05, Students’ t test. However, the lower HbF levels observed in males in our study were higher than that reported by Adekile et al. (8.9 vs 7.5 %), that might have masked the effect of HbF in our patients. This bias is further highlighted by the higher value of mean Hb in our males (9.7 vs. 8.4 g/dl). Thus, the gender effect observed by Adekile et al^18^ may indeed be related to the effect of HbF.

### Hydroxyurea therapy

Hydroxyurea therapy is known to influence hemoglobin level and hemolytic marker expression. In this cohort of 136 SCA subjects, 61 [28-males, 33- females] patients were on stable HU therapy. However, there were no significant differences in the hemoglobin levels or markers of hemolysis.

### Effect of Age

Age is an important risk factor in the incidence of gallstone formation in SCA. In the Jamaican Cohort Study involving patients routinely followed from the age of 5 years with ultrasound, 7% had cholelithiasis before the age of 5 years and 15% by the age of 10 years.^19^ In contrast, in our population we did not find any gallstone below the age of 10 years. However, it cumulatively increased with age, reaching 70% by the third decade in this cohort.

### Effect of UGT1A1 polymorphism

We first excluded the potential coinfluence of alpha thalassemia and β^s^ genotype on *UGT1A1* (Table 3a and 4a) since these factors can significantly modify the substrate load on *UGT1A1*. As shown in Table 2 the *UGT1A1* polymorphism influences only, but highly significantly, the serum total bilirubin level. Comparison of *UGT1A1* group 1 versus group 2 or 3 emphasizes the importance of the dominant effect of (TA)_5_ over others regarding total bilirubin level in a background where the values of indicators of the degree of hemolysis were comparable between groups. In the absence of assessment of unconjugated bilirubin, we like other reports, assume that the increment in total serum bilirubin observed in group 2 and 3 (as opposed to group 1) is the consequence of relative bilirubin conjugation deficiency. However, Chaar V et al^9^ had observed that only unconjugated bilirubin concentration and not conjugated bilirubin concentration differed between *UGT1A1* groups. Nevertheless, the observation by Kaplan et al, that *UGT1A1* polymorphism may influence total serum bilirubin, by both increasing the heme catabolism (by a mechanism yet to be identified) as well as by diminishing the rate of conjugation merits further consideration.^20^

These parameters indicate clearly that the Arab-Indian β^s^ type SCA patients exhibit a lower degree of hemolysis as compared to African β^s^ type SCA patients. However, there was no difference in the rate of gallstones between these two groups. Here again, there is a disassociation between the degree of hemolysis/hyperbilirubinemia and the prevalence of gallstones. The statistically significant higher mean age of randomly selected Arab-Indian SCA patients may highlight their milder clinical course (presentation at a later age) as compared to African β^s^ SCA group (see Table 4b). It is important to note as the rate of gallstone formation increases with age.

### Effect of alpha thalassemia

Conflicting data have been reported in the literature regarding the role of alpha thalassemia in bilirubinemia and cholelithiasis in SCA.^1^,^4^,^11^,^13^ In our study, although the coexisting alpha thalassemia diminished the hemolysis in a gene dose-dependant manner (low LDH and reticulocyte numbers and higher Hb), it did not influence the rate of gall stone formation. Despite the possible limitation of our study due to lack of exploration of non-deletional alpha thalassemic alleles (not so rare in this region) the αα/αα genotype group differs significantly from the -α/ -α genotype group for all the hemolysis–related features (except gall stone formation) making less likely the presence of non-deletional alpha thalassemic alleles in the latter.^21^

### Influence of β^s^ genotype

Very few studies had explored the influence of different β^s^ genotypes on serum total bilirubin level and cholelithiasis for the very fact that the analyzed SCA patients were haplotypewise homogeneous excepting a study by Adekile *et al*^18^. These authors by studying SCA patients from North America, essentially with various combinations of African haplotypes, failed to note any influence on serum bilirubin.

Our study clearly indicates that the Arab-Indian β^s^ SCA patients exhibit a lower degree of hemolysis as compared to African β^s^ SCA patients. However, there was no difference in the rate of gallstones between these two groups. Here again, there is a disassociation between the degree of hemolysis/hyperbilirubinemia and the prevalence of gallstones. The statistically significant higher mean age of the selected Arab-Indian SCA patients may highlight their milder clinical course (presentation with gallstones at a later age) as compared to African β^s^ SCA group (Table 4b). This observation is important since the rate of gallstone formation increases with age.

To our knowledge, the present study is the first report to compare the influence of African and Arab Indian haplotypes on serum bilirubin and cholelithogenesis. Intriguingly, despite statistically significant differences in HbF, Hb, Reticulocyte count and bilirubin (Table 4b) between these sickle cell groups, there was no difference in the incidence of gall stone. Since the prevalence of alpha thalassemia among these sickle cell genotype groups were not statistically different, the differences in the hemolysis–related features and serum bilirubin are very likely due to high HbF expression in the Arab-Indian group.

Thus our report is the first comparing the relative influence of Arab-Indian and African β^s^ genotypes on the bilirubin and cholelithiasis in SCA patients.

## Figures and Tables

**Table 1a:** Gender effect on hematological/biochemical parameters [mean±SD] and incidence of Cholelithiasis in Omani SCA patients [n=136]

Sex	Male (N=65)	Female(N=71)
Age, Yrs	22.7±9.2	23.5±8.5#
Hb, g/dl	9.7±1.6	9.1±0.9#
Absolute Reticulocyte Count, × 10**^9^**/L	233±84	231±84#
Hb F, %	8.9±6.7	10.1±6.6#
Total Serum Bilirubin, μmol/L	50.9±36.7	42.2±44#
Gallstone n, (%)	34, (52.3)	37, (52.1)#
On Hydroxyurea n, (%)	28, (43)	33, (46.5) #

# - Not significant, comparing males with females, Student’s t test

**Table 1b:** Effect of age decade on mean hematological/biochemical parameters and incidence of cholelithiasis in Omani SCA patients [n=136]

Age decade in years	<10 (n=7)	10–19 (n=31)	20–29 (n=71)	30–39 (n=17)	40–49 (n=9)	50–59 (n=1)
Mean Age ±SD	6.9±1.7	15.6±2.8	24.6±2.6	33.1±2.5	42.5±2.9	51.7
Hb, g/dl	8.7±0.7	9.1±1.2	9.6±1.3	10.6±1.4	9.8±1.0	8.8
HbF, %	12.7±7.3	10.3±12.4	8.1±5.8	9.6±8.1	11.6±7.7	15.7
Absolute Reticulocyte Count, × 10**^9^**/L	267±98	217±91	218±86	197±88	199±113	212
Total Serum Bilirubin, μmol/L	42.1±23	53.7±63	41.8±32.1	30.5±18.5	30.4±16.5	24.3
Gallstone by decade group	None	51.6%	54.9%	70.5%	44.4%	None

**Table 2:** Biochemical/Hematological parameters [mean±SD] and gall stone status in ultrasonography assessed SCA patients stratified for *UGT1A1* (TA)_n_ genotype [n=133].

*UGT1A1* (TA) n genotype n=133	Group 1 n=4	Group 2 n=113	Group 3 n=16	p value [1 v/s 3]	p value [1 v/s 2]	p value [2 v/s 3]
Total Serum Bilirubin, μmol/L	22.1±7.6	44.6±40.2	73.5±66.2	**8.1**×**10^−7^**#	**0.002**#	**0.05**#
Serum LDH, IU/L	583±267	404±149	431±202	NS#	NS#	NS#
Hemoglobin, g/dl	9.06±0.38	9.4±1.3	9.8±1.7	NS#	NS#	NS#
Absolute Reticulocyte Count, × 10**^9^**/L	259±81	231±83	240±91	NS#	NS#	NS#
HbF, %	7.9±5.08	9.68±8.8	9.5±6.5	NS#	NS#	NS#
Gallstones, n =71, (%)	2(50)	60(53.1)	9(56.3)	NS#	NS#	NS#

Group 1: *UGT1A1* genotypes (TA)_5_/(TA)_6_, (TA)_5_/(TA)_7_; Group 2: *UGT1A1*genotypes (TA)_6_/(TA)_6_, (TA)_6_/(TA)_7_; Group 3: *UGT1A1*genotypes (TA)_7_/(TA)_7_,(TA)_7_/(TA)_8_, (TA)_8_/(TA)_8_. **p<0.05** – Significant, NS-p>0.05, *Student’s t test;

# Chi Square test

**Table 3a:** Distribution of *UGT1A1* genotypes in the SCA patients grouped by the α-Globin status [n=105].

	αα/αα, n=11	α/αα, n=46	-α/-α, N=48	p value
***UGT1A1*** Group 1, n=4 (%)	**2(50)**	**0(0)**	**2(50)**	NS#
***UGT1A1*** Group 2, n=89 (%)	**8(9)**	**40(45)**	**41(46)**	NS#
***UGT1A1*** Group 3, n=12 (%)	**1(8)**	**6(50)**	**5(42)**	NS#

NS - p>0.05,;

# Chi Square test

**Table 3b:** Biochemical/Hematological parameters [mean±SD] and gall stone status in SCA patients grouped by the α-Globin status [n=105].

	αα/αα, n=11	αα/αα, n=11	-α/-α, N=48	p value [αα/αα v/s -α/-α]	p value [αα/αα v/s -α/αα]	p value [-α/αα v/s -α/-α ]
Total Serum Bilirubin, μmol/L	64.85±34.8	53.4±53.3	30.1±16.7	**6.5**×10^−7^*	NS*	**9.2**×**10^−5^**#
Serum LDH, IU/L	528±160	528±160	376±161	0.008*	NS*	**0.02**#
Hemoglobin, g/dl	8.3±1.0	9.36±1.2	9.7±1.27	0.00026*	0.003*	NS#
Absolute Reticulocyte Count, × 10**^9^**/L	314±64	234±82	217±74	**3.2**×10^−5^*	0.001*	NS#
HbF, %	11.2±3.5	10.0±4.1	7.6±3.1	0.006*	NS*	NS#
Gallstones, n (%)	9(82)	26(57)	23(48)	NS*	NS*	NS#

p<0.05 –Significant, NS -p>0.05,

* Student’s t test;

# Chi Square test

**Table 4a:** *UGT1A1* and Alpha genotype in the Arab Indian v/s Non-Arab Indian SCA homozygous haplotypes [n=77].

	Homozygous Arab- Indian[n=20]	Homozygous Non-Arab-Indian[n=57]	p value
*UGT1A1*(TA)_5_/(TA)_6_,(TA)_5_/(TA)_7_;n=4, (%)	1(25)	3(75)	NS#
*UGT1A1*(TA)_6_/(TA)_6_,(TA)_6_/(TA)_7_; n=58,(%)	14(24)	44(76)	NS#
*UGT1A1*(TA)_7_/(TA)_7_,(TA)_8_/(TA)_8_; n=15,(%)	5(33)	10(67)	NS#
Alpha genotype :αα/αα, n=10,(%)	2(20)	8(80)	NS#
Alpha genotype :-α/αα, n=33,(%)	11(32)	22(68)	NS#
Alpha genotype : -α/ -α, n=34,(%)	7(21)	27(79)	NS#

NS – p >0.05;

# Chi Square test

**Table 4b:** Demographic, Biochemical and hematological parameters [mean±SD], and Gallstones in the Arab Indian v/s Non-Arab Indian SCA homozygous haplotypes [n=77].

	Homozygous Arab- Indian[n=20]	Homozygous Non-Arab-Indian[n=57]	p value
Age, yrs	27.1±9.8	21.51±8.7	0.005*
Total Serum Bilirubin, μmol/L	34.7±19.4	49.9±18.9	0.02*
Serum LDH, IU/L	347±61	466±63	0.05*
Hemoglobin, g/dl	10.1±0.97	9.25±1.3	0.001*
Absolute Retic Count, ×10^9^/L	187.9±97	246±86	0.018*
HbF, %	15.72±5.1	8.3±6.5	0.00001*
Gallstones, n=39,(%)	10(50)	29(51)	NS#

NS – p >0.05; p<0.05 – Significant;

* Students’t test,

# Chi Square test.
